# Nutritional Status and Metabolism in Celiac Disease: Narrative Review

**DOI:** 10.3390/jcm12155107

**Published:** 2023-08-03

**Authors:** Aleksandra Mędza, Agnieszka Szlagatys-Sidorkiewicz

**Affiliations:** 1 Department of Pediatrics, Pediatric Gastroenterology, Allergology and Nutrition, Copernicus Hospital, Nowe Ogrody 1-6, 80-803 Gdansk, Poland; 2 Department of Pediatrics, Pediatric Gastroenterology, Allergology and Nutrition, Medical University of Gdańsk, 80-210 Gdansk, Poland; agnieszka.szlagatys-sidorkiewicz@gumed.edu.pl

**Keywords:** celiac disease, malnutrition, metabolism, nutrients, gluten-free diet

## Abstract

This review summarizes findings from studies assessing the nutritional status of patients with celiac disease (CD). Malnutrition, including over- and undernutrition, may be present in CD, both at diagnosis and while under treatment. Underweight and growth retardation in children, which mostly reflect malabsorption as a consequence of intestinal inflammation, are not a rule. Clinical presentations of CD can vary widely, and each manifestation has its own characteristics. Evaluating various nutritional parameters can be beneficial for CD patients and may improve health outcomes by facilitating an accurate definition of dietary needs and the development of a balanced diet that not only focuses on eliminating gluten but also provides adequate nutrients, alters metabolism, and reduces the risk of other disorders developing. The cornerstone of CD therapy is a gluten-free diet (GFD), which improves nutritional status, but even on a GFD, features of malnutrition may be present. Additionally, overweight and obesity may occur in patients on a GFD, with typical metabolic consequences.

## 1. Introduction

Celiac disease (CD) is an inflammatory disease that occurs after ingestion of gluten as a result of complex interactions between genetic, immunological, and environmental factors. The estimated prevalence varies depending on the diagnostic process, with 1.4% individuals affected worldwide if serologic tests are used and 0.7% individuals worldwide based on biopsy [1]. The loss of gluten tolerance leading to the onset of the disease can occur at any time in life as a consequence of other triggers in addition to gluten, such as gastrointestinal infections, medications, α-interferon, or surgery [2]. Classical presentations include chronic diarrhea, failure to thrive, abdominal distension, and malnutrition due to intestinal malabsorption. Non-classical presentations include gastrointestinal and extra-intestinal symptoms, such as bloating, constipation, chronic fatigue, headaches, abdominal pain, and osteoporosis. The literature contains descriptions of hematologic abnormalities in CD, such as microcytic or macrocytic anemia, thrombocytosis, coagulopathy, thromboembolism, or thrombocytopenia [3]. Some of them are related to malabsorption and deficits of iron, vitamins B6 and B12, and folate. This diversity of presentations and the associated severity of CD may be one of the reasons for variations in the nutritional status seen at diagnosis. Clinicians should remain alert to patients with T1DM, in whom CD prevalence can be up to twenty times higher than in the general population [4]. The link between those two conditions is the human leukocyte antigen (HLA) system, where they share common characteristics, HLA-DQ8 and HLA-DQ2, key genetic risk factors in both [5]. The fact that up to 83.78% of children with T1DM do not have gastrointestinal symptoms, even if they meet the serological criteria for a CD diagnosis, is a challenge for specialists [6]. Patients with both celiac disease and type 1 diabetes mellitus (T1DM) require comprehensive management to address the multiple co-occurring morbidities.

The only treatment for celiac disease is a lifelong gluten-free diet (GFD) that restores the intestinal mucosa and improves body mass, bone mineral density [7], and reproductive health [8]. Maintaining a strict yet nutritionally balanced GFD is a lifelong challenge. Therefore, each patient should be provided with professional dietary support to improve their adherence to the diet and to avoid both deficiencies and excess intake of macronutrients, vitamins, and minerals [9,10].

This paper aims to characterize the nutritional status of celiac disease patients at diagnosis and after the introduction of a GFD, with its metabolic consequences. We will also discuss metabolic differences occurring in CD patients.

In this narrative review, a PubMed search was conducted using the following search terms: “celiac disease” AND “nutrients”, ”metabolism”, ”nutritional status”, “malnutrition”, “overnutrition”, “obesity”, ”Gluten Free Diet”, “deficiency”, “deficits”. The search strategy restricted papers to those written in English and with full text available. The article search was carried out by one author (A.M.), who screened the titles and abstracts of the selected records. Authors reviewed the publications and selected those of the best quality, going first to papers containing a meta-analysis or systematic review, followed by original studies and other kinds of papers.

## 2. Indices of Nutritional Status of Celiac Disease Patients at Diagnosis

“Malnutrition” is a common term for conditions caused by excesses, deficiencies, or imbalances in a person’s intake of energy and/or nutrients. These include overnutrition, undernutrition, or micronutrient-related malnutrition, which pose a real global problem. Undernutrition, which includes wasting (low weight-for-height), stunting (low height-for-age), and underweight (low weight-for-age), affects 462 million people worldwide. In contrast, 1.9 billion adults are overweight, obese, and at risk of diet-related noncommunicable diseases (such as heart disease, stroke, diabetes, and some cancers) [11].

Patients with celiac disease present with malnutrition, as defined earlier. Existing studies report varying levels of particular deficits. Examples are presented in Table 1.

### 2.1. Nutrient Deficits at Diagnosis

Untreated patients present nutrient malabsorption related to villous atrophy of the intestinal mucosa. This is the reason for poor absorption of amino acids and fats, as well as for deficiencies in micronutrients, including calcium; iron; zinc; copper; vitamins A, D, E, and K; folate; and pyridoxine. In the study conducted by Wierdsma, almost all CD patients (87%), including those who were obese, had at least one vitamin or mineral value below the lower reference limit. In that paper, more than 20% of the patients reported taking vitamin and mineral supplements before the diagnosis of CD was made. These deficiencies were independent of the Marsh stratum, age, and body mass index (BMI) category [17]. Other research evaluating children with celiac disease did not find associations between micronutrient status and fecal gluten immunogenic peptide (GIP), height, socioeconomic status, or gastrointestinal symptoms [19]. Deficits were observed, as presented in Table 1, but the authors also noted that all children had blood levels of vitamin B2, folate, and vitamin B12 within the normal reference intervals. They were assessed as low risk for deficiencies of magnesium and vitamin C.

A poor vitamin status in celiac disease has been linked with increased risk of thrombosis [32,33]. The pathophysiologic connection has been explained by disturbance in homocysteine metabolism, which requires vitamin B6, vitamin B12, and folate. The interruption in the metabolic path of homocysteine results in hyperhomocysteinemia, which is a procoagulant state [34].

Malabsorption of calcium and vitamin D3 with a chronic inflammatory state affects bone health and may result in osteopenia or osteoporosis in celiac disease. Duodenal villous atrophy, through malabsorption, is a determinant factor for low bone mineral density (BMD) in adult-onset patients [35]. It has been presented that the Marsh histopathological stage can be a predictor of low BMD and the risk of developing osteoporosis [36]. In the mentioned study, patients with a higher Marsh histopathological stage had lower serum calcium and higher parathyroid hormone levels compared to mild cases at diagnosis. Therefore, it is recommended to measure calcium, alkaline phosphatase, and vitamin D at CD diagnosis to assess bone health. Moreover, it is necessary to perform bone mineral density measurement with the dual X-ray absorptiometry (DEXA) scan in adults, not later than the age of 30–35 years, preferably earlier in patients with risk factors for low BMD [2]. Previous studies report that 30–60% of newly diagnosed patients present low BMD and 18–35% osteoporosis [37]. Therefore, calcium and vitamin D3 should be supplemented in celiac disease patients with documented low serum levels, those with loss of BMD, or those who cannot achieve adequate intake via diet to prevent osteoporosis and fractures.

### 2.2. Growth and BMI at Diagnosis

Chronic undernutrition categorized as stunting, which is characterized by low height-for-age, is associated with celiac disease. Growth failure in terms of height may be the earliest sign of celiac disease [38]. A recent meta-analysis concluded that approximately 1 in 14 patients with all-cause short stature and 1 in 9 patients with idiopathic short stature has biopsy-confirmed CD [39].

The BMI, easily obtained from simple height and weight measures, has become a popular method for the assessment of both undernutrition and excess adiposity. However, one must be aware of its limitations and the fact that it does not allow for accurate estimating of body composition. The BMI does not provide an indication of individual fractions of body composition and proportions between them. It varies substantially with age and sex, especially during the pubertal growth spurt. Therefore, in children, it should be used not as an absolute value but as a relative value, with a reference to centile curves [40]. The BMI has high specificity but low sensitivity for detecting excess adiposity and fails to identify over a quarter of children with excess body fat percentage [41]. However, it is a widely used, easy, and cheap method. A meta-analysis of the BMI in adults and children with CD showed that the mean BMI of celiac disease patients at presentation is significantly lower than that of controls. Although most patients were within the normal BMI range (67%), surprisingly, 14% were overweight, 6% were obese, and only 13% were underweight at diagnosis. In subgroup analysis, the prevalence of normal weight was significantly higher in children than in adult patients (71% vs. 61%, respectively) [12]. More data are presented in Table 1.

The overnutrition aspect in celiac disease at diagnosis is surprising. Indeed, while its main hallmark is enteropathy, clinical presentations are diverse. One study reported that almost half of adult CD patients with a BMI over 25 kg/m^2^ are affected with the subclinical or silent type of celiac disease [42]. It is extremely important for clinical practice that obesity, present also in childhood [43], not exclude the diagnosis of CD in order to prevent delays in treatment. Factors connected with the risk and prevention of metabolic syndrome and cardiovascular disease should be taken into account in that group. The authors of the systematic review and meta-analysis [44] concluded that celiac disease is associated with a modestly increased risk of cardiovascular disease, attaining statistical significance only for stroke, with limited evidence. They reported that CD is not associated with myocardial infarction and cardiovascular deaths. In a different study, Huang [45] did not find a causal contribution of CD as such to ischemic stroke, large artery stroke, cardioembolic stroke, small vessel stroke, coronary heart disease, myocardial infarction, angina, heart failure, atrial fibrillation, or venous thromboembolism risk. He performed two-sample Mendelian randomization using large-scale Genome-Wide Association Study (GWAS) summary data. This suggests that other factors, rather than CD itself, may contribute.

## 3. Nutritional Issues Modulated by a GFD

### 3.1. Nutrients and a GFD

A GFD involves certain dietary restrictions that create a real challenge for people with CD in the implementation of all their nutritional recommendations for a balanced lifelong diet. The main focus is on gluten avoidance, but it lacks attention to the nutritional quality of the diet. The composition of gluten-free products is unsatisfactory, and they are not enriched with micronutrients. Thus, the content of vitamin D3; folate; vitamins B1, B2, B3, and B12; iron; zinc; magnesium; and calcium in gluten-free products is low [46,47]. Moreover, the bioavailability of iron, calcium, manganese, and zinc is inhibited by phytates contained in gluten-free products [48,49]. Therefore, the levels of these nutrients in patients on a GFD may be lower, depending on individual diet quality. The literature suggests that nutrient deficiencies in CD patients may be related more to the nutritional inadequacy of GFDs rather than malabsorption [50], so deficits need to be monitored long term and supplemented, where indicated. However, primary deficits of vitamins B1, K, and E and ferritin at diagnosis are reversible on a GFD [19].

Interesting data from Spain [51,52] indicated that the composition of a GFD consumed by women and men with CD does not differ much from the diet of the general population. Most micronutrient deficiencies in men were not directly related to a gluten-free diet and concerned the entire population. In women, vitamin D, iron, and iodine compared poorly with suggested dietary references. Fat and protein consumption was above recommendations, and fiber intake was poor in both men and women. In men, the dietary profile in CD patients was as unbalanced as in the controls, and 38% of men included in the study [52] consumed calories in excess of their energy expenditure. This male subgroup included an alarming 38.1% of overweight and obese individuals, although the prevalence of overweight and obesity was still lower than in the control population. Fat mass measurements using bioelectrical impedance analysis (BIA) indicated that 52% of men in the study [52] had excessive adiposity, while less than 6.5% of women were overweight, and there were no cases of obesity [51].

### 3.2. BMI on a GFD

Authors of a meta-analysis assessing the risk of obesity on a gluten-free diet observed some increases in the mean BMI but concluded that a GFD does not increase the risk of developing overweight or obesity [12]. They observed significant increases in the mean BMI in celiac disease patients on a GFD in comparison with the BMI at diagnosis. However, the percentages of patients in the overweight and obese categories remained similar to those observed at disease presentation, at 13% vs. 14% and 7% vs. 6%, respectively. The data demonstrated that 9% of the entire study population moved to a higher BMI category. This percentage was higher in the adult subgroup than in children (12% vs. 6%). In contrast, 20% of the study population moved from the overweight/obese category to the underweight/normal category, with the percentage significantly higher in the pediatric versus the adult population (34% vs. 8%). The authors noted that there was a high risk of bias and that suboptimal quality studies were included in the meta-analysis.

Explanations for the increase in weight on a GFD may be hypothetical compensatory hyperabsorption after the restoration of intestinal mucosal function with the GFD [53] or a poor composition of the GFD.

### 3.3. Impact of a GFD and Its Composition on Health Status

The elimination of gluten has numerous benefits in terms of alleviating discomfort in patients with celiac disease and reducing the risk of complications, thanks to seroconversion of pathogenic antibodies and intestinal mucosa healing. However, eliminating gluten from the diet often disturbs the proportion of nutrients consumed, leading to metabolic disorders. A well-balanced GFD is not easy to follow on a daily basis.

#### 3.3.1. Metabolic-Associated Fatty Liver Disease

A GFD has been associated with altered nutrient intake and cardiovascular and metabolic syndrome risk both in children and in adults, with its hepatic hallmark, nonalcoholic fatty liver disease (NAFLD). NAFLD has been recently given a new definition, namely “metabolic-associated fatty liver disease” (MAFLD) [54]. The crucial factors here are gluten-free products with a high sugar content, including glucose syrup, starch, and flour derived from rice. This results in a high glycemic index, predisposing individuals on a GFD to the development of insulin resistance [55]. Tortora compared the metabolic status of patients at diagnosis and after 1 year on a GFD [56]. In his group, 2% of patients fulfilled the diagnostic criteria for metabolic syndrome at diagnosis, while 29.5% fulfilled the criteria after 1 year on a GFD. After a year, he noted that there was a significant increase in the BMI, waist circumference, blood pressure (BP), and blood glucose levels. Further results of Ciccone’s investigation are similar, as he observed the prevalence of metabolic syndrome in 3.24% of CD patients at diagnosis and 14.59% on a GFD [57], with the highest rates of metabolic changes found after 5 to 10 years on a GFD.

It has been observed that more than a third of CD patients adhering to a GFD have concurrent NAFLD/MAFLD, which accounts for a three-fold increase in risk compared to the general population [58]. The factors independently related to MAFLD are not only the body mass index, total cholesterol, and triglycerides but also dietary habits, with an increase in the consumption of packaged gluten-free foods (PGFF) consisting mainly of products categorized as “bread and bakery”, “salty convenience”, and “sweet convenience” [59]. The authors hypothesized that the mechanisms contributing to the steatogenesis include, first, increased amounts of carbohydrate and fats absorbed from those products arriving at the liver through portal flow and, second, short-chain fatty acids interacting with the gut microbiota that may dysregulate the production of acetate and propionate (two regulators of de novo lipogenesis in the liver). Nestares et al. warn against high consumption of ultra-processed food, which may result in a worse inflammatory profile (higher macrophage inflammatory protein-1α, soluble superoxide dismutase-1, and 15-F2t-isoprostanes) [9].

The problem of poor composition of gluten-free foods also affects bread, whose gluten-free varieties have a lower protein and a higher fat content than gluten-containing bread, with the dietary fiber content highly variable in different countries. Moreover, label information is at significant variance from the data obtained through the chemical analysis for fiber and other nutritional components [60].

#### 3.3.2. Brain and Neurological State

Among the many symptoms of celiac disease, there are also neurological conditions. They can be improved with the introduction of a GFD. If headaches coexist in the disease manifestation, a strict GFD can lead to total resolution in up to 75% of patients [61]. It has been presented that diet modulates changes in the brain caused by CD, which can be characterized using neuroimaging. Brain imaging showed a greater rate of cerebellar gray matter atrophy in the antibody-positive group compared to the antibody-negative group in a 7-year follow-up study [62]. Authors analyzed antibodies against endomysium, transglutaminase 6 (TG6), and transglutaminase 2 (TG2). They concluded that patients with CD who do not adhere strictly to a GFD and stay serological positive are at risk of developing ataxia and have a significantly higher rate of cerebellar atrophy.

Additionally, it has been described that a strict diet can improve neuroimaging results in patients with gluten-sensitivity-related disorders. The results of magnetic resonance spectroscopy, conducted on adults with cerebellar manifestations and gluten sensitivity with or without enteropathy, changed before and after a GFD. Authors noted an increase in the NAA/Cr ratio in the cerebellar vermis in 98% of cases on a strict GFD [63]. The NAA/Cr ratio is a metabolic marker reflecting the functional status of neurons and axons in the brain. Its decrease indicates neuronal or axonal loss or dysfunction [64].

In children with drug-resistant epilepsy and a diagnosis of celiac disease, a GFD in combination with antiseizure medications appears to reduce the number of epileptic seizures [65].

Following gluten elimination, normalization of the EEG recording is observed in children with celiac disease who have previously been found to have unexplained EEG abnormalities [66].

#### 3.3.3. Intestines

Adherence to a strict GFD can reduce intestinal symptoms, restore the histology of the small bowel architecture, restore a normal villous-to-crypt (V/C) ratio, and reduce inflammation; however, the epithelium maintains some stigmata of the disorder, such as an increased number of CD3+ and TCRγδ+ cells [67]. An incomplete recovery of the intestinal mucosa was observed in a group of patients after 1 year on a GFD who had more severe mucosal damage, higher antibody values, and more signs of malabsorption at diagnosis [68]. Additionally, the recovery effect depends on the time of gluten elimination and the age of patients, with possible total recovery in 100% of children but not in adults [69,70,71].

The benefits of using a GFD also include protection against intestinal cancer, such as enteropathy-associated T cell lymphoma (EATL), to which CD patients are at risk. Strict adherence seems to be the only possibility of preventing a subset of this rare but aggressive disease [72].

#### 3.3.4. Bone Health

Bone metabolism abnormalities are the element of the gluten-dependent clinical picture of CD. The currently approved pharmacologic therapeutics for the treatment of adults or the prevention of osteoporosis are bisphosphonates, but they are still off-label for children. However, studies prove that a GFD alone is effective for the increase in BMD values [37]. This occurs thanks to the healing of lesions of the intestinal mucosa when calcium is absorbed properly, and the earlier bone demineralization decreases. Even after only 6 months of strict adherence to a GFD, the BMD of children improved [73], and in those with osteopenia, their z-score returned to normal [74]. A GFD should be balanced with proper calcium and vitamin D3, controlled by a dietitian. It is extremely important for children, whose growth is incomplete, and calcium intake is an essential determinant of the bone mass peak.

### 3.4. GFD in Patients with CD and Type 1 Diabetes Mellitus

A recent meta-analysis and systematic review of research concerning children with comorbid type 1 diabetes mellitus (T1DM) and asymptomatic CD, on a GFD for least 12 months, revealed that the GFD had no significant effect on the BMI or glycosylated hemoglobin. The analysis reported an improved lipid profile (high-density lipoprotein (HDL) cholesterol, total cholesterol, and triglyceride) and an increase in hemoglobin and serum iron levels following strict adherence to a GFD for at least 6 months in this particular group. Adherence to a GFD in patients with T1DM and CD might have a protective effect on the development of diabetic reno- and retinopathy [75].

## 4. Metabolism

There are abnormalities in fat and carbohydrate metabolism in CD. It was noted that carbohydrate oxidation is preferential, probably as a result of lipid malabsorption and high carbohydrate intake, in untreated individuals with celiac disease. These data come from a study in which only patients with the classic form of CD were enrolled. Moreover, high carbohydrate oxidation rates correlate positively with fecal lipid loss in untreated patients [76]. A GFD increases lipid use with the restoration of the intestinal mucosa, and this results in a significant increase in body fat stores, even after only 1 year of treatment [77].

The alteration in lipid metabolism is also reflected in serum lipid profiles, with an observed link between low plasma cholesterol and celiac disease [78]. The literature contains reports of low total cholesterol and low high-density lipoprotein cholesterol (HDL-C) in patients at diagnosis [78,79,80]. A systematic review [81] identified consistent findings across studies on alteration in the blood lipid profile after dietary treatment, which included an increase in total cholesterol and HDL.

Lipid profile changes have been linked to decreased intestinal synthesis of ApoA-I during active celiac disease and decreased dietary lipid absorption. The restoration of the lipid profile in CD patients after GFD treatment may be explained by an increase in both ApoA-I secretion by intestinal cells and the fat mass, which positively correlates with the increase in HDL-C in CD patients on a GFD [82].

In the area of lipid profile alterations, Auricchio et al. [83] explored serum phospholipid profiles, as they were looking for molecules that can be potential CD biomarkers. They identified a specific serum phospholipid signature that predicts the onset of celiac disease in HLA at-risk infants before the appearance of antibodies specific for CD in the serum and before any clinical symptoms, even before gluten introduction into the diet at 4 months. In a different study, Sen et al. [84] found that children who develop CD have increased triacylgycerols (TGs) of a low carbon number, double-bond count plasma levels, and decreased phosphatidylcholine and cholesterol ester levels at 3 months of age compared to healthy controls. These differences were exacerbated with age and were not present at birth in the samples of cord blood. These studies suggest that a dysregulation of lipid metabolism may be associated with the development of CD and that it occurs in the first months of life, before exposure to gluten in a diet. These findings prove the importance of metabolomic analysis in increasing the understanding of celiac disease and improving diagnostic and therapeutic strategies [85]. By analyzing metabolites, it is possible to gain insight into the pathophysiology of CD and better understand the impact of treatment on patients. Metabolomics studies hold particular significance in unraveling the complexities of multifactorial and multisystemic disorders, such as celiac disease. More studies need to be conducted in this area.

To summarize, lipid and carbohydrate metabolism is modified in patients with celiac disease but also in individuals with the potential to develop it.

## 5. Conclusions

Features of malnutrition are observed in patients with celiac disease, at diagnosis and after treatment, but each individual may present different aspects, varying from under- to overnutrition, with or without specific deficits. Careful observation of nutritional status parameters by professional healthcare providers may prevent metabolic diseases and nutrient deficiencies. Metabolism of fats and carbohydrates in celiac disease patients differs from that in healthy individuals. Remarks for clinicians are presented in Table 2.

## Figures and Tables

**Table 1:** Prevalence of particular nutritional features in CD at diagnosis.

Indices of Malnutrition	Prevalence of Deficits in CD at Diagnosis		Comments
	Adults%	Children%	
Underweight BMI * < 18.5 kg/m^2^	13 [12]	13 [12]	In this paper [12], pediatric body mass categories were defined using BMI centiles, adjusted for age and sex, or z-scores, depending on the definition used by the authors.
Overweight BMI 25 up to 29.9 kg/m^2^	20 [12]	9 [12]	
Obesity BMI > 30 kg/m^2^	10 [12]	3 [12]	
Height	Males: mean 168.5 ± 8.6 in CD vs. 171.3 ± 7.2 cm in healthy controls [13]		A short stature in adults correlates with the duration of symptoms before diagnosis [14].
	Females: mean 154.8 ± 10.58 in CD vs. 157.8 ± 7.2 cm in healthy controls [13]		
		Height SD * score below −2.0 in 30% of CD patients; in 76%, weight-for-height below the median [15]	Histopathological abnormalities (Marsh grade) do not correlate with the final height in CD [13]. The mean height SD score showed increasing growth retardation in the year before diagnosis, relatively quick catch-up in growth in the year following diagnosis, and complete catch-up after 2–3 years of treatment [15]. If a child is diagnosed post-puberty, their chances for catch-up growth are much lower [16].
Vitamin A deficiency	7.5 [17] Males: 11.8 Females: 5.6	Range from 0 [18] to 20 [19] up to 32.7 [20]	Clinical manifestations of deficits in serum retinol include night blindness, conjunctival dryness, and keratomalacia.
Vitamin D3 deficiency	4.8 [17]–59 [21]	24 [19]–61.5 [20]	The 25(OH)D3 level in treated patients returns to normal independently of supplementation [21,22]. Vitamin D3 affects bone health; however, the literature is inconclusive on vitamin D3 deficits impacting bone mass density in CD [23,24].
Vitamin E deficiency		Range from 0 [20] to 2.4 [18] up to 88 [19]	
	In both adults and children: 100 [25]		
Vitamin K deficiency		0 [20]–21 [19] Deficits are mostly detected using the prolonged prothrombin time.	Malabsorption of vitamin K leads to impaired hepatic synthesis of coagulation factors II, VII, IX, and X, resulting in the prolongation of coagulation assays. Clinical manifestations such as hemorrhage are rare [26].
Vitamin B6 deficiency	14.5 [17] Males: 25 Females: 9.5	12.5 [19]	
Vitamin B12 deficiency	19 [17] Males: 22.2 Females: 17.3		Histologically and serologically atrophic (corpus) gastritis was ruled out [17]. Deficits are present despite the fact that vitamin B12 absorption occurs in the terminal ileum, which is free from typical mucosal lesions. The reason for this deficit is unclear.
Folate deficiency	20 [17] Males: 28.5 Females: 15.4		Underweight patients have a slightly higher serum folic acid concentration than patients with normal weight or overweight patients [17].
Calcium deficiency	Rarely detected. Analysis of calcium metabolism, not calcium levels, should be mandatory.		Despite calcium malabsorption, hypocalcemia is rarely detected, due to secondary hyperparathyroidism [27] and the increase in the active metabolite of vitamin D3, 1,25-dihydroxyvitamin D, which regulates the calcium balance [28].
Low hemoglobin	12–85 [29]	10–15 [30]	Iron-deficiency anemia is one of the most common extra-intestinal manifestations at diagnosis in adult CD patients [16,31]. Iron is predominantly absorbed in the duodenum, which is the main portion of the bowel affected by CD.
Low ferritin	46.2 [17] Males: 30 Females: 51.7	79 [19]	Serum ferritin values were lower when the villous atrophy Marsh grade was higher [17].
Zinc deficiency	66.7 [17] Males: 62.5 Females: 70.8	33 [19]	

* BMI: body mass index; SD: standard deviation.

**Table 2:** Remarks for clinicians dealing with celiac disease patients.

Remarks for Clinicians
Overweight or obesity does not exclude celiac disease.
Assess for metabolic syndrome, cardiovascular risk, and the possibility of MAFLD.
Refer your patients for a DEXA scan to control bone mass density. Refer your patients to a dietitian for adequate calcium and vitamin D3.
Refer your patient to a dietitian for GFD adherence control, balanced diet composition, and weight maintenance counseling. Recommend nutrient supplementation, if needed.
Patients with DM1 should be checked for celiac disease.
Patients with chronic neurological symptoms or drug-resistant epilepsy should be screened for celiac disease.

## Data Availability

No new data were created in this study. Data sharing is not applicable in this article.
